# Host Shift Speciation of the Ectomycorrhizal Genus *Suillus* (Suillineae, Boletales) and Biogeographic Comparison With Its Host Pinaceae

**DOI:** 10.3389/fmicb.2022.831450

**Published:** 2022-03-30

**Authors:** Rui Zhang, Xiao-fei Shi, Pei-gui Liu, Andrew W. Wilson, Gregory M. Mueller

**Affiliations:** ^1^Guangdong Provincial Key Laboratory for Plant Epigenetics, Longhua Bioindustry and Innovation Research Institute, College of Life Sciences and Oceanography, Shenzhen University, Shenzhen, China; ^2^Key Laboratory of Optoelectronic Devices and Systems of Ministry of Education and Guangdong Province, College of Optoelectronic Engineering, Shenzhen University, Shenzhen, China; ^3^Program in Plant Biology and Conservation, Northwestern University, Evanston, IL, United States; ^4^Key Laboratory of Biodiversity and Biogeography, Kunming Institute of Botany, Chinese Academy of Sciences, Kunming, China; ^5^Plant Conservation Science, Chicago Botanic Garden, Glencoe, IL, United States; ^6^Sam Mitchel Herbarium of Fungi, Denver Botanic Garden, Denver, CO, United States

**Keywords:** ancestral range, diversification rate, fossil calibration, host specificity, multigene phylogeny

## Abstract

*Suillus* is a genus of ectomycorrhizal fungi associated almost exclusively with Pinaceae. Lack of sample collections in East Asia and unresolved basal phylogenetic relationships of the genus are the major obstacles for better understanding the *Suillus* evolution. A resolved phylogeny of *Suillus* representing global diversity was achieved by sequencing multiple nuclear ribosomal and protein coding genes and extensive samples collected in East Asia. Fungal fossils are extremely rare, and the Eocene ectomycorrhizal symbiosis (ECM) fossil of *Pinus* root has been widely used for calibration. This study explored an alternative calibration scenario of the ECM fossil for controversy. Ancestral host associations of *Suillus* were estimated by maximum likelihood and Bayesian Markov chain Monte Carlo (MCMC) analyses, inferred from current host information from root tips and field observation. Host shift speciation explains the diversification of *Suillus* major clades. The three basal subgenera of *Suillus* were inferred to be associated with *Larix*, and diverged in early Eocene or Upper Cretaceous. In the early Oligocene or Paleocene, subgenus *Suillus* diverged and switched host to *Pinus* subgenus *Strobus*, and then switched to subgenus *Pinus* four times. *Suillus* subgenus *Douglasii* switched host from *Larix* to *Pseudotsuga* in Oligocene or Eocene. Increased species diversity occurred in subgenus *Suillus* after it switched host to *Pinus* but no associated speciation rate shifts were detected. Ancestral biogeographic distributions of *Suillus* and Pinaceae were estimated under the Dispersal Extinction Cladogenesis (DEC) model. Ancestral distribution patterns of *Suillus* and Pinaceae are related but generally discordant. Dispersals between Eurasia and North America explain the prevalence of disjunct *Suillus* taxa.

## Introduction

The ectomycorrhizal symbiosis (ECM) is a common relationship between plants and fungi responsible for exchanging carbohydrates, mineral nutrients, and water (Landeweert et al., 2001; Nehls et al., 2010). Extant ectomycorrhizal fungi have evolved independently from over 80 saprotrophic ancestral lineages (Tedersoo and Smith, 2013, 2017; Martin et al., 2016).

The majority of ECM fungi are generalists, whereby one ECM fungal species can form an association with a wide variety of plant families (Bruns et al., 2002; Kennedy et al., 2003; Smith et al., 2009). However, a subset of ECM fungi is host-specific, i.e., they are associated with particular plant taxonomic groups. In contrast, their host plants are commonly generalists with the ability to recruit a broad spectrum of ECM fungal taxa (Ishida et al., 2007; Krpata et al., 2008; Deslippe et al., 2011; Leski and Rudawska, 2012). Species in the ECM genus *Suillus* have exceptionally high host fidelity to specific trees within the pine family (Pinaceae), with varying specificity from genera down to species (Thiers, 1975; Kretzer et al., 1996; Nguyen et al., 2016).

The evolutionary mechanisms explaining host specificity between symbiotic fungi and host plants include host shift speciation, coevolution, and cospeciation (Vienne et al., 2013). Host shift speciation has been confirmed in many ECM fungal lineages, including *Strobilomyces*, *Leccinum, Hebeloma, Pisolithus*, and *Laccaria* (Aanen et al., 2000; Martin et al., 2002; Bakker et al., 2004; Sato et al., 2017; Wilson et al., 2017). Diversification after host switching has also been documented in *Strobilomyces*, *Leccinum*, and *Hebeloma*, providing the ecological advantages of adapting to novel hosts and niches (Aanen et al., 2000; Bakker et al., 2004; Sato et al., 2017).

Being host-specific, most *Suillus* species are restricted to the Northern Hemisphere along with their Pinaceae hosts (Nguyen et al., 2016). Studies have shown that *Suilloid* taxa might have facilitated the invasion of Pinaceae into the Southern Hemisphere (Dickie et al., 2010; Hayward et al., 2015; Policelli et al., 2019). Diversity of *Suillus* species is severely underestimated in East Asia, with a significant lack of sampling hindering further biogeographic analyses (Wu et al., 2000; Mueller et al., 2001). Despite current efforts to discover novel species, the contemporary richness of the *Suillus* diversity in East Asia remains unevaluated (Verma and Reddy, 2015; Shi et al., 2016; Zhang et al., 2017).

This study compares the evolutionary relationships and distribution patterns of *Suillus* and their hosts Pinaceae throughout history. Multiple nuclear protein-coding genes of *Suillus* were collected, given that previous phylogenies based solely on ribosomal rRNA sequences could not resolve basal relationships of the genus (Kretzer et al., 1996; Wu et al., 2000; Mueller et al., 2001). To fill the sampling gap, many *Suillus* specimens were collected from East Asia, covering the geographic distributions of Pinaceae hosts, including approximately 26 putative undescribed species. We addressed three specific questions in this study, including (1) Can cospeciation or host shift speciation explain the diversification of *Suillus* major lineages? (2) Are geographic distribution patterns between *Suillus* and its host plants congruent? (3) Are the extensive speciation or diversification rate shifts driven by *Suillus* host switches or geographic range variation?

## Materials and Methods

### Specimens and Molecular Data Collection

*Suillus* specimens from North American and European herbaria were sampled for DNA extraction. To fill the geographic sampling gap, intensive field collections were conducted over 10 years in East Asia covering Pinaceae distribution ranges (Farjon, 1990, 2005). A list of 103 *Suillus* specimens representing approximately 86 species used in this study is given in Supplementary Table 1.

DNA extraction, PCR and cycle sequencing are as previously described (Zhang et al., 2017). For nuclear ribosomal rRNA internal transcribed spacers 1 and 2, the 5.8S rRNA gene and parts of the 28S rRNA genes, primers ITS-1F and ITS-4 were used (White et al., 1990; Gardes and Bruns, 1993). For some specimens with DNA degradation, amplification was carried out with internal primers ITS-2 and ITS-3 (White et al., 1990). For the nuclear partial 28S large subunit rRNA genes, primers LROR and LR5 were used (Vilgalys and Hester, 1990). For amplification of the nuclear protein coding translation elongation factor EF1-alpha (*TEF1*) partial gene, the following primers were used: primers *TEF1*-983f and *TEF1*-2212r, with additional internal primer *TEF1*-1567r (Rehner and Buckley, 2005), and one newly designed *Suillus* specific internal primer *TEF1*-Sintf (5′- TYR CAC AGC ATG MCA TGG TA -3′). For amplification of the nuclear protein coding RNA polymerase II largest subunit (*RPB1*) partial gene, the following primers were used: primers *RPB1*-Af and *RPB1*-Cr (Stiller and Hall, 1997; Matheny, 2005), with additional internal primer *RPB1*-Int2.2f (Binder et al., 2010) and *RPB1*-Int2.1r (Frøslev et al., 2005). For amplification of the nuclear protein coding RNA polymerase II second largest subunit (*RPB2*) partial gene, the primers were: *RPB2*-6F and *RPB2*-7.1r (Matheny et al., 2007), and newly designed *Suillus* specific internal primers *RPB2*-SintR (5′- CTC CRT CNT CNT CGC GRT AA -3′) and *RPB2*-SintF (5′- CAC GAC CRG CRT CYG TGT AY -3′).

### Phylogenetic Analyses

An ITS phylogeny of *Suillus* was generated for identifying operation taxonomic units and for more information on host, distribution range and diversity. *Rhizopogon abietis*, *Rhizopogon ochraceisporus*, and *Truncocolumella citrina* were chosen as outgroups for the ITS dataset (Binder and Hibbett, 2006). Initial alignment for the ITS sequences was obtained in Mesquite 2.75 with manual adjustments (Edgar, 2004; Maddison and Maddison, 2011).

Phylogenetic tree of ITS sequences was inferred from Maximum likelihood (ML) and Bayesian methods performed on the CIPRES computing facility (Miller et al., 2015). Bootstrap analyses of ML were performed using RAxML 8.0.0 HPC2 on XSEDE with 1000 bootstrap replicates (Stamatakis, 2014). Bayesian phylogenetic analysis was performed using MrBayes 3.2.6 (Ronquist and Huelsenbeck, 2003; Ronquist et al., 2012). Number of substitution types Nst was set at 6, with 2 runs, 4 chains per run, each run searching for 1,000,000 generations sampling every 1000th generation. The first 10% of the sampled Bayesian trees of the analysis was discarded as the burnin. For convergence diagnosis, the estimated sample size (ESS) was above 200.

A supermatrix dataset was assembled with four loci including 28S, *TEF1*, *RPB1*, and *RPB2*. For the supermatrix dataset, each sample was selected from the ITS phylogeny to represent a unique haplotype. ITS sequences and introns of protein coding genes were not included because of their high level of sequence variability and poorer resolution at deeper nodes. Before concatenation, significant topological incongruence was evaluated among single gene phylogenies of 28S, *TEF1*, *RPB1*, and *RPB2* using a cutoff of ≥ 70% maximum likelihood bootstrap (MLB) support and ≥ 0.98 Bayesian posterior probabilities (BPP). No major conflicts were detected for the inter-species evaluation. *Rhizopogon nigrescens* and *Gomphidius roseus* were chosen as outgroups for the multigene dataset (Binder and Hibbett, 2006).

Partitioning strategy and molecular models were searched using PartitionFinder V1.1.1 (Lanfear et al., 2012). The greedy algorithm was used to explore all nucleotide substitution models available under the Bayesian information criterion (BIC). Codon positions of protein coding genes were regarded for the partitioning analyses. Phylogenetic trees of the supermatrix were constructed with RAxML and Bayesian methods as described for the ITS phylogeny. Partitioning schemes and substitution models were set for the RAxML analysis under GTRGAMMA with 1000 bootstrap replicates. For the BI analysis, MrBayes 3.2.6 was implemented with the partitioned supermatrix and substitution models suggested by PartitionFinder (Ronquist et al., 2012).

### Reconstructing Ancestral Host Associations

Current host associations of *Suillus* species were identified from global environmental samples, field observations and literature references. To be more conservative on host identifications, mycological references containing taxonomic uncertainties were not included. Host information for a certain *Suillus* species is documented if root tip samples were in the same OTUs defined by ≥ 70% MLB or ≥ 0.98 BPP in the ITS phylogeny. Field observations can provide reliable host information for Asian *Suillus* because almost each *Pinus* and *Larix* species grows in separate habitat (Farjon, 1990, 2005). Hosts of *Suillus* were classified to the generic level for *Larix* and *Pseudotsuga*, and to the subgeneric level for *Pinus*.

To reconstruct ancestral host associations for *Suillus*, ML and Bayesian Markov chain Monte Carlo (MCMC) analyses were conducted in BayesTraits v2.0 (Pagel and Meade, 2006). MCMC analyses were run for 1.01 × 10^6^ iterations, sampling every 1,000, with the first 10,000 iterations discarded as a burn-in. The most probable host with a common ancestor was inferred if the acceptance rate is between 20–40% when the chain is at convergence.

### Current Distributions of *Suillus* and Pinaceae

Collection site data were obtained from herbarium labels for *Suillus* taxa. Current ranges of *Suillus* species were also inferred from the global ITS phylogeny including environmental samples. Introduced taxa were excluded from the biogeographic analyses, e.g., *Suillus luteus* associated with introduced *Pinus sylvestris* in North America and *S. lakei* with introduced *Pseudotsuga menziesii* in Europe.

The current distributions of Pinaceae species were limited to their natural ranges (Farjon, 1990, 2005). *Pinus*, *Larix*, and *Pseudotsuga* distribution data across the world were also compiled from the global biodiversity information facility database (GBIF^1^.

### Ancestral Range Estimation

To estimate ancestral ranges we used the Dispersal Extinction Cladogenesis (DEC) model of Lagrange implemented in R package BioGeoBEARS (Ree and Smith, 2008; Matzke, 2014). The founder-event dispersal parameter “j” was introduced for DEC assuming a cladogenetic event (Matzke, 2014).

The extant distributions of *Suillus* and Pinaceae were divided into four biogeographic units: (1) ENA: Eastern North America, east of the Rocky mountains, encompassing Canada to Florida; (2) WNA, Western North America, west of the Rocky mountains, encompassing Alaska through Central America; (3) EUA, encompassing Europe, Northern China and Central Asia; (4) In, Indo-Pacific, encompassing subtropical Southern China, Southeast Asia and the western Himalayas. We also refer the combined EUA and Indo-Pacific as Eurasia. Events including cladogenesis, anagenesis, and vicariance were interpreted based on the most probable range for each ancestral species.

### Time-Scaled Phylogenies of *Suillus* and Pinaceae

A two-step calibration procedure was conducted following previous examples (Renner, 2005; Wilson et al., 2012, 2017). Step one of the BEAST analysis used two fungal fossils to calibrate the phylogeny of 37 Agaricomycete taxa, including 19 *Suillus* taxa. Taxonomic groups were defined in BEAUti including Agaricales, Boletales, Boletineae, Boletinus, Sclerodermatineae, “Marasmioid” fungi and Suillineae. *Suillus* taxa from five subgenera represent the genus. Two fossils in Agaricomycetes were utilized for calibration in step one. *Archaeomarasmius leggetti* from mid-Cretaceous amber of New Jersey resembles the extant genera *Marasmius* and *Marasmiellus* (Hibbett et al., 1997). *Archaeomarasmius leggetti* was regarded as the most recent common ancestor (MRCA) of *Marasmius rotula* and *Mycena amabillisima* and was calibrated at 90 Ma (million year ago) with a mean of 10 using a lognormal distribution (Renner, 2005; Wilson et al., 2012, 2017). The second fossil used for calibration is an ECM root of *Pinus* preserved in the Princeton chert of British Columbia in the early Eocene (*c.* 47.8 Ma) (Lepage et al., 1997; Pigg and Devore, 2016). The ECM fossil was used to calibrate the node for the Suillineae, including *Suillus, Rhizopogon*, and *Gomphidius*, and was calibrated using a lognormal distribution with an initial value 50 Ma and a log (mean) set 25. The same set of fossil calibrations was carried out in previous studies of Agaricomycetes (Floudas et al., 2012; Wilson et al., 2012, 2017). The Princeton chert fossil does not provide information on sporocarp morphology but the *Pinus* host is known. This study explores an alternative scenario: the fossil could be at clade(s) of *Suillus* associated with *Pinus*. Step two calibration inferred calibration points of major clades from step one to estimate divergence dates of the comprehensive *Suillus* phylogeny.

We used BEAUti v.1.8.2 to create XML files that incorporated the calibration priors, partitioning schemes and Bayesian parameters for analysis in BEAST v1.8.2 (Drummond et al., 2012). Bayesian parameters included a GTR + I + G model, Yule process speciation, and an uncorrelated lognormal relaxed clock model. The Bayesian Markov-chain Monte Carlo (MC^3^) analysis was run for 30 million generations, sampling every 1000th tree. Each analysis was run two times. The first 10% of the trees were removed as the burn-in and the remaining trees were combined using LogCombiner v1.8.2. A summary tree was produced using TreeAnnotator v1.8.2 (Drummond et al., 2012). Convergence, burn-in, means, medians and 95% highest posterior densities (HPDs) for nodes of interest were examined from BEAST logfiles using Tracer v1.6.0. Maximum clade credibility trees from the two steps were created using TreeAnnotator (BEAST package) and to summarize the posterior samples of trees produced by BEAST.

The calibrated phylogeny of *Pinus* was pruned from a published phylogeny of conifers (Leslie et al., 2012), which was generated from two nuclear genes (18S and a phytochrome gene, *PHYP*) and two chloroplast genes (*mat*K and *rbc*L). It included 84% of *Pinus* global diversity. Fossils *Larix altoborealis*, *Picea burtonii*, and *Tsuga swedaea* within Pinaceae were used to calibrate the phylogeny (Leslie et al., 2012).

### Diversification Rate Shifts

Lineage-through-time (LTT) plots were conducted in the R package APE for *Suillus.* For better comparison, *Suillus*/*Pinus* lineages and genus *Pinus* were plated on the same LTT plot (Paradis et al., 2004; Leslie et al., 2012).

To reveal speciation rate variations in the phylogeny of *Suillus*, Bayesian analysis of Macroevolutionary Mixtures (BAMM) v2.0 was utilized (Rabosky, 2014; Rabosky et al., 2014). BAMM reconstructs branch-specific evolutionary rates and allows rates to vary through time and among lineages. Without prior knowledge of the number and location of distinct regimes of diversification rates, BAMM simulates a posterior distribution of shift configurations on phylogenetic trees. The priors for the BAMM run was simulated by BAMMtools (Rabosky et al., 2014). Our sampled 66 OTUs are about 72% of the *c.* 92 known global *Suillus* OTUs. The employed incomplete sampling of 60% estimated unknown *Suillus* diversity from unsampled Pinaceae hosts. BAMM was run under the reversible-jump MCMC method with 10 million generations for the calibrated phylogenies of Pinaceae and *Suillus*. The first 10% was discarded as burn-in and the convergence was checked in coda (Plummer et al., 2008). BAMMtools visualized the output of BAMM to generate the mean phylorate plot and the 95% credible set of macroevolutionary rate configurations (credible shift sets). If zero rate shift was detected in the phylogeny, the expected number of shifts was not adjusted in BAMMtools to avoid type I error (Rabosky et al., 2014).

## Results

### Phylogenetic Analyses

A total of 393 sequences were generated (93 ITS, 89 28S, 90 *TEF1*, 61 *RPB1*, 60 *RPB2*), and 98 sequences were acquired from GenBank (94 ITS, 1 28S, 1 *TEF1*, 1 *RPB1*, 1 *RPB2*) (Supplementary Table 1). The ITS dataset was 834 bp in total length with 313 parsimony informative sites. We added 26 new OTUs representing new species from China and other studies (Verma and Reddy, 2015; Shi et al., 2016). RAxML and Bayesian analyses provided congruent results for the ITS phylogeny (Supplementary Figure 1). Deep relationships of the ITS phylogeny remain unresolved (Supplementary Figure 1). Host and geographic information was inferred from the ITS phylogeny and was applied to the *Suillus* species level (Supplementary Figure 1 and Supplementary Table 1). Matrices and phylogenetic trees are available in Treebase (number S21096^2^).

For the multigene phylogeny, we sampled 66 OTUs (72% of known *Suillus* OTUs). Four OTUs contained multiple geographic representatives: *Suillus brevipes*, *Suillus ampliporus*, *Suillus flavidus*, and *Suillus spectabilis*. For the subsequent multigene analysis, taxa were selected from the ITS phylogeny to evenly represent the taxonomic and geographic diversity within *Suillus* (Supplementary Figure 1). The total length of the supermatrix was 3914 bp with 858 informative sites. The supermatrix excluding introns was 3138 bp with 626 informative sites and was partitioned as: (1) 28S and the first and second codon positions of *TEF1*, *RPB1*, and *RPB2*, with GTR + I + G as the best model; (2) the third codon positions of *TEF1*, *RPB1*, and *RPB2* with GTR + G as the best model.

**FIGURE 1:**
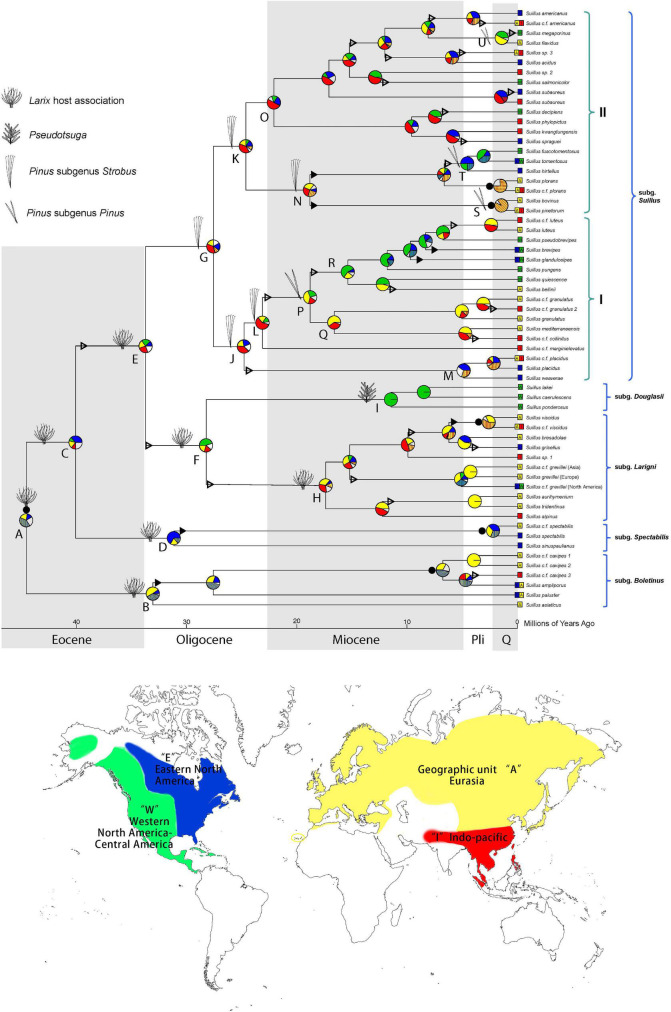
Historical biogeography of *Suillus* estimated by DEC + J model in BioGeoBEARS. Pie diagrams at each node denote geographical units or combination of units occupied by ancestral taxa. Geographic units are represented by different colors. Combined units are shown by hatching colors or by lettering. Width of pie wedges refers to the probability of that geographic unit or combination of units. White wedges indicate the sum of the units (or combined units) with individual probabilities < 15%. Triangles on branches denote “jump” dispersal events (founder effect cladogenesis), solid triangles indicate range expansion dispersal events (anagenesis), and solid circles by pie diagrams represent vicariant speciation events. Ancestral host associations were annotated at the nodes. Letters by key nodes are discussed in the text. Current geographic ranges of terminal taxa are indicated by colored boxes and lettering. Parentheses indicate the subgenera and section as annotated.

Phylogenies based on the supermatrices resolved the basal relationships of *Suillus* (Supplementary Figure 2), with five subgenera resolved and supported (Figure 1 and Supplementary Figure 2). The subgenus *Boletinus* (node D) was basal and sister to all other subgenera. In contrast, the subgenus *Spectabilis* (node F) was sister to a monophyletic clade (node G) containing all remaining subgenera. The subgenus *Larigini* was sister to the subgenus *Douglasii*, and the common ancestor of the two was sister to the subgenus *Suillus*. We recommend that two new monophyletic sections be recognized for the subgenus *Suillus*—I and II (Supplementary Figure 4; node M and L).

### Evolutionary History and Host Associations of *Suillus*

Step one calibration under the Suillineae scenario at node A (Supplementary Figure 3A) indicated that the crown age of *Suillus* was *c*. 40.2 (31.9–50.8) Ma. Subgenera *Boletinus*, *Spectabilis*, and *Larigini* are all associated with *Larix*. Subgenus *Douglasii* switched host to *Pseudotsuga* at a stem age of *c.* 28.2 (median, 22.4–34.5) Ma in the mid-Oligocene (Supplementary Figure 4; node H). Another independent host switch, from *Larix* to *Pinus*, occurred in the late Eocene to early Oligocene, with a stem age of 33.7 (29.0–39.7) Ma (Supplementary Figure 4; node G). Ancestral host reconstruction revealed that the *Pinus* association initially occurred in *Pinus* subgenus *Strobus*, and later the subgenus *Suillus* independently switched to subgenus *Pinus* four separate times. The earliest switch to the subgenus *Pinus* was in section I at a stem age of 23.1 (18.6–27.8) Ma (Supplementary Figure 4; node N), leading to a relatively prosperous clade associated with subgenus *Pinus*. All other host switches to subgenus *Pinus* were in section II, among which the *Suillus pinetorum* and *S. bovinus* clade switched first in 18.8 (14.4–23.8) Ma (Supplementary Figure 4; node O). At node Q, a clade consisting of *S. tomentosus*, *S. fuscotomentosus*, and *S. hirtellus* switched hosts in the late Miocene at 6.6 (4.3–9.5) Ma. Lastly, at node R, the *Suillus flavidus* and *S. megaporinus* clade switched hosts in the late Miocene at 8.1 (5.0–11.6) Ma.

*Suillus* species switched to *Pseudotsuga*, while *Pinus* did not revert to the ancestral *Larix* association. Host associations with both subgenera of *Pinus* are reported for *S. flavidus* and *S. acidus* but should be further verified. For the North American *S. subaureus*, an initial association with *Pinus* subgenus *Strobus* is required for transferring into *Quercus* in later developmental stage, therefore *Quercus* was not included in the BayesTraits analysis (Lofgren et al., 2018). Further, *Suillus sinuspaulianus* was also excluded from the host reconstruction due to the uncertainty of its host association (Pomerleau and Smith, 1962; Kretzer et al., 1996).

### Alternative Calibration Scenario of the Ectomycorrhizal Symbiosis Fungal Fossil

Ancestral host association supported the *Suillus-Pinus* association in the subgenus *Suillus* (Clade I, Supplementary Figure 4). The *Pinus* root ECM fossil can be regarded as the subgenus *Suillus*, thus provide an alternative calibration scenario (Lepage et al., 1997). The ECM fossil under the *Suillus-Pinus* scenario was calibrated at the stem of subgenus *Suillus* (Supplementary Figure 3B, node G). The new calibration revealed an earlier origin of Agaricomycetes in 299.2 (237.7–360.7) Ma versus 159.6 (112.8–262.9) Ma in the first scenario. It also indicated that Boletales originated 279.5 (220.2–337.5) Ma, much earlier than the estimations of scenario one and other studies (Supplementary Table 2). Further, it found that *Suillus* diverged from *Rhizopogon* in the Upper Cretaceous, and the crown age of *Suillus* was 71.1 (60.6–85.7) Ma (Supplementary Figure 3B and Supplementary Table 2). *Suillus* switched from *Larix* to *Pinus* in Paleocene 54.3 (50.4–62.2) Ma at node G; from *Larix* to *Pseudotsuga* in Eocene 46.5 (36.2–56.3) Ma at node H (Supplementary Figure 3B and Supplementary Table 2). Inclusive *Suillus* tree and ancestral host reconstruction under the *Suillus-Pinus* scenario are provided in Supplementary Figures 3, 5.

### Biogeographic History of *Suillus* and Pinaceae

The ancestors of *Larix* and *Suillus-Larix* were circumboreally distributed (Figure 1 and Supplementary Figure 6). Extant *Larix* species have distinct ranges within each continent. *Suillus* has dispersed across the North American and Eurasian continents more frequently than *Larix*. Disjunct pairs of *Suillus* were discovered from Oligocene to as recent as the *Suillus paluster* and *S. ampliporus* species complexes (Figure 1). The ancestor of *Pseudotsuga* was circumboreally distributed and diverged into current species with disjunct distributions (Supplementary Figure 6). Three *Suillus* species were associated with *Pseudotsuga menziesii* in Western North America (WNA, Figure 1, clade K).

Distinct biogeographic patterns were found in *Pinus* but not in *Suillus* associated with *Pinus*. For the subgenus *Strobus*, *Pinus* section *Parrya* was limited to WNA (Supplementary Figure 6, clade H). In contrast, no *Suillus* specimens were found in association with *Pinus* section *Parrya.* The *Pinus* section *Quinquefoliae* originated in Eurasia and dispersed to the New World, with the most recent lineages returning to Eurasia (Supplementary Figure 6, clade I). *Suillus* that switched host from *Larix* to sect. *Quinquefoliae* were reconstructed in the circumboreal region (Figure 1, clade I), with their current distributions influenced by both vicariance and inter-continental dispersal events. Prevalent disjunct Eurasian and North American taxa include *S. placidus*, *S. kwantungensis-S. spraguei*, *S. decipience-S. phylopictus*, *S. subaureus*, and *S. americanus*. For the subgenus *Pinus*, section *Pinus* was distributed in Eurasia, with exception of *Pinus resinosa* in Eastern North America (ENA), and *P. tropicalis* in WNA. While the section *Trifoliae* was mainly distributed in WNA before dispersing to ENA three times for a quarter of its species. For *Suillus* associated with subgenus *Pinus*, section *Suillus* was originally located in the circumboreal region. Clade S retained a basal Eurasian species, dispersed to North America, and then dispersed back to Eurasia. Compared with *Pinus*, North American lineages in clade S shifted host from section *Pinus* to section *Trifoliae* and then to *Pinus* concomitant with inter-continental dispersal. Originating from a circumboreal ancestor associated with five-needle pines, clade O diverged into a few Eurasian species associated with *Pinus* section *Pinus*, and another clade T associated with *Pinus* section *Trifoliae.* Finally, clade U includes two disjunct species: *Suillus megaporinus* from WNA and *S. flavidus* from North Eurasia.

### Speciation Rate Shifts in *Suillus* and Pinaceae

The LTT plot reveals the constant accumulation of *Suillus* lineages through time (Supplementary Figure 7). In the phylorate plot, diversification rate shifts were not detected for *Suillus* (Supplementary Figure 8). The second calibration scenario does not influence this result. Diversification rates (average range 0.12–0.14) were homogenous across *Suillus* lineages but varied through geological time along the phylogenetic tree branches.

## Discussion

### Lacking Cospeciation Patterns Between *Suillus* and Pinaceae

**FIGURE 2:**
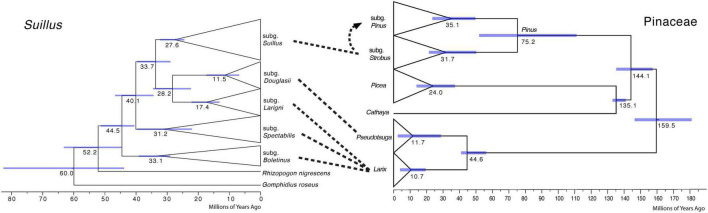
Comparison of calibrated phylogenies of *Suillus* and host Pinaceae genera. The phylogeny of *Suillus* is on the left, where terminal taxa are collapsed into triangles and only subgenera are shown. The phylogeny of Pinaceae is on the right, where host genera and two subgenera of *Pinus* are annotated. The phylogeny of Pinaceae is referred from Leslie et al. (2012). Dashed lines between the two phylogenies indicate host associations. Subgenus *Suillus* first established symbiosis with subgenus *Strobus* and then switched to subgenus *Pinus* as shown by a dashed arrow.

Cospeciation patterns were not identified between the *Suillus* subgenera and Pinaceae genera (Figure 2). Phylogenetic topologies were distinct between *Suillus* and Pinaceae. Basal subgenera of *Suillus* were all associated with *Larix*. Subgenera *Larigni* and *Douglasii* were sister clades, as were their hosts *Larix* and *Pseudotsuga*. Yet, given that only one species of *Pseudotsuga* was the host for subgenus *Douglasii*, this pattern was not congruent (Murata et al., 2013; Wen et al., 2014). *Picea* and *Cathaya* are not documented hosts for *Suillus*. *Picea* has been reported as one of the putative host genera for a species within the subgenus *Spectabilis*, but further studies are required to confirm this (Pomerleau and Smith, 1962). *Pinus* diverged into two subgenera, while subgenus *Suillus* diverged into two clades (I and II), which are not congruent with the host subgenera. If cospeciation had occurred between *Suillus* and Pinaceae, the phylogeny of *Suillus* should mirror that of Pinaceae in both topology and evolutionary time.

### Host Shift Speciation of *Suillus*

Host shift speciation explained the evolutionary history of *Suillus* (Figure 1 and Supplementary Figure 4). To effectively adapt to a novel host, *Suillus* populations need to expand, accumulate variations, and experiment over sufficient time to successfully inoculate (Vienne et al., 2013). Host recognition of *Suillus* involves plant pathogenic pathways, and host switching might trigger plant defensive responses (Liao et al., 2016). *Suillus* basal lineages were associated with *Larix* for *c*. 20 million years before switching to the subgenus *Strobus*. Further, the subgenus *Suillus* was associated with the subgenus *Strobus* for *c*. 10 million years before switching to the subgenus *Pinus*. All these time periods were significantly longer under the second ECM fossil calibration scenario.

*Suillus* phylogeny did not mirror the phylogeny of Pinaceae, otherwise, host switching from *Larix* to a more closely related *Pseudotsuga* would have occurred before the switching to the *Pinus* subgenus *Strobus*, i.e., *Picea* and *Cathaya* would not have been skipped (Figure 2). The possibility cannot be excluded that extinction may help to explain the current host associations of *Suillus*, as *Suillus* could have switched to a now-extinct ancestor of *Pseudotsuga*, remaining only with *Pseudotsuga menziesii* (Murata et al., 2013; Wen et al., 2014).

A one-way direction of ECM fungal host subgenera level switching was first observed in *Suillus*, and this could be a unique trait in host-specific ECM fungi. After *Suillus* switched hosts from *Larix* to *Pseudotsuga* and the subgenus *Strobus*, they did not reverse to *Larix*. The same phenomenon is observed in the subgenus *Suillus*, whereby its host switched from the *Pinus* subgenus *Strobus* to the subgenus *Pinus*. It is plausible that the directional host switches reduce interspecific competition for ecological niches. *Suillus* species are pioneer ECM fungi for establishing plant seedlings and are generally not dominant in these underground communities (Gardes and Bruns, 1996; Zhou and Hogetsu, 2002; Policelli et al., 2019). Due to this ecological limitation, reducing interspecific competition is evolutionarily advantageous. Interspecific competition could also originate from another source: if different *Suillus* species inoculate the same host plant, they might compete for the same molecular pathways for symbiotic recognition and establishment (Liao et al., 2016).

Another question worth exploring is how *Larix* became the ancestral host for the genus *Suillus*. *Truncocolumella*, the sister genus of *Suillus*, is associated with *Pseudotsuga* and *Tsuga* (Zeller, 1940; Smith and Singer, 1959; Binder and Hibbett, 2006). The next closely related genus *Rhizopogon*, which is associated with *Pinus*, *Picea*, *Pseudotsuga*, *Larix*, *Tsuga*, *Abies*, and *Picea* of Pinaceae, as well as several Angiosperm genera (Molina and Trappe, 1994; Binder and Hibbett, 2006). Both host shift speciation and extinction may explain the basal association with *Larix* in the genus *Suillus*.

### Uncertainty of the Ectomycorrhizal Symbiosis Fossil for Calibration

As a result of the ephemeral existence and soft tissue of fungal sporocarps, fungal fossils are extremely rare (Berbee and Taylor, 2010). The Eocene ECM fossil of Princeton chert has been widely used in mycological calibrations under the Suillineae scenario (Floudas et al., 2012; Wilson et al., 2012, 2017; Aime et al., 2018; Krah et al., 2018). But the application of this fossil for fungal calibration is controversial for the following reasons. The fossil was identified as a *Pinus* root tip with Suillineae ectomycorrhizae (Lepage et al., 1997). The fossil provided no sporocarp characters so its identity was uncertain. If host association of the ECM fossil is regarded as a prominent character, cautions must be made as other alternative calibration scenarios exist. We tried an alternative calibration scenario of the fossil in the *Suillus-Pinus* lineage. Yet different scenarios of the ECM fossil in the *Rhizopogon-Pinus* lineages have not yet been explored. As *Suillus* does not strictly cospeciate with *Pinus*, it is not applicable to compare the evolutionary history of *Pinus* and *Suillus* to find the preferred scenario.

### Comparing Biogeographic Histories of *Suillus* and Pinaceae

The crown age of Pinaceae remains controversial, even using different fossils to calibrate (Wang et al., 2000; Lin et al., 2010; Leslie et al., 2012; Lu et al., 2014; Gernandt et al., 2016; Ran et al., 2018). As the original date of *Suillus* is still debated, rigid comparison between *Suillus* and *Pinaceae* referring to paleoclimate and geographic histories was refrained. Instead, general biogeographic patterns were compared with emphasis on the disjunct taxa of Eurasia-ENA and Eurasia-WNA. The biogeographic histories of *Suillus* and Pinaceae are generally discordant, as discussed below.

Consistent with other studies, the ancestor of *Larix* and *Pseudotsuga* was reconstructed to be within the circumboreal region (Supplementary Figure 6; Semerikov et al., 2003; Wei and Wang, 2003, 2004). However, other studies have recognized three biogeographic clades of *Larix* with phylogenetic supports: two Eurasian clades and one North American clade (Semerikov et al., 2003; Wei and Wang, 2003, 2004). No distinct geographic clades of *Suillus* associated with *Larix* were found in our study (Figure 1). Dispersions of *Larix* and *Suillus* were likely through the Bering land bridge (BLB) and the North Atlantic land bridge (NALB) (Wang and Ran, 2014; Jiang et al., 2019). North American and East Asian clades of *Pseudotsuga* are supported with phylogenetic data (Wei et al., 2010). Three *Suillus* species were in association with *Pseudotsuga menziesii* in WNA. With no ancient *Suillus* lineages found in the WNA, the host switching to *Pseudotsuga* could have assisted the *Suillus* dispersal to WNA. *Suillus* taxa were anticipated in Asian *Pseudotsuga* trees but were not found after extensive environmental sampling (Murata et al., 2013; Wen et al., 2014).

Vicariance plays a major role in shaping the *Pinus* biogeography, as all major clades of *Pinus* have distinct ranges (Eckert and Hall, 2006; Hao et al., 2015; Gallien et al., 2016). Long-distance dispersal across North America and Eurasia occurred approximately 3–5 times in *Pinus*. In contrast, inter-continental dispersal events influenced the evolution of the *Suillus* subgenus *Suillus*. North American and Eurasian disjunct taxa, arising in different geological epochs, were prevalent in subgenus *Suillus*. WNA is enriched with two *Pinus* sections and half of the *Pinus* diversity. The radiation of *Pinus* in WNA was shaped by the complex climatic history and orogeny of the Rocky Mountains and the Mexican highlands (Mastretta-Yanes et al., 2015; Antonelli et al., 2018; Hagen et al., 2019). In contrast, major clades of subgenus *Suillus* were not limited within biogeographic divisions, and the diversity of *Suillus* in WNA was relatively low. In addition, *Suillus* sporocarps were not yet identified from *Pinus* section *Parrya*, though *Suillus* was putatively reported from *Pinus edulis* root tip samples (Patterson et al., 2019). *Suillus* might have shifted to the WNA *Pinus* relatively recent in geological time; thus, climatic history and orogeny did not have the same effects on *Suillus*. Alternatively, the ability for *Suillus* to frequently disperse over long distances could have blurred their geographic boundaries.

### Diversification Rate Shifts of *Suillus*

The BAMM analysis detected no speciation rate shifts within *Suillus*. Reported diversification rate shifts in fungi usually involve hyper diversified lineages initiated with a key innovation or migration into a significantly different environment (Kraichak et al., 2015; Sánchez-Ramírez et al., 2015; Wilson et al., 2017). Yet, *Suillus* host switches do not involve fundamental changes in living habit or adapting to very different environments. Host *Pinus* and the *Suillus* subgenus *Suillus* follow independent evolutionary trajectories. Overall, *Suillus* are almost absent from the Pinyon pines. Establishing associations with the subgenus *Pinus* lagged until the early Miocene or Eocene; therefore, *Suillus* likely missed the opportunity to diversify extensively in concert with subgenus *Pinus*.

## Data Availability Statement

The original contributions presented in the study are included in the article/Supplementary Material, further inquiries can be directed to the corresponding authors.

## Author Contributions

GM, P-GL, and RZ designed the study. RZ and XF-S performed the experiments. RZ and AW analyzed the data. RZ wrote the manuscript. All authors contributed to the article and approved the submitted version.

## Conflict of Interest

The authors declare that the research was conducted in the absence of any commercial or financial relationships that could be construed as a potential conflict of interest.

## Publisher’s Note

All claims expressed in this article are solely those of the authors and do not necessarily represent those of their affiliated organizations, or those of the publisher, the editors and the reviewers. Any product that may be evaluated in this article, or claim that may be made by its manufacturer, is not guaranteed or endorsed by the publisher.

## Footnotes

1
https://www.gbif.org

2
https://treebase.org/treebase-web/
