# Supplementary material for: Host Shift Speciation of the Ectomycorrhizal Genus Suillus (Suillineae, Boletales) and Biogeographic Comparison With Its Host Pinaceae
Source: Front Microbiol. 2022 Mar 30;13:831450. doi: 10.3389/fmicb.2022.831450 (PMC9009389; doi:10.3389/fmicb.2022.831450)
Supplement: Supplementary file 1 [file Data_Sheet_1.PDF]

Supporting Information

Article title: Host shift speciation of ectomycorrhizal genus *Suillus* (Suillineae, Boletales) and biogeographic comparison with host Pinaceae

Authors: Rui Zhang, Xiao-fei Shi, Pei-gui Liu, Andrew W. Wilson and Gregory M. Mueller

The following Supporting Information is available for this article:

Table S1 Herbaria specimens and molecular sequences used in this study. GenBank sequences are underlined. Host information is included in this table inferred from environmental samples containing root tip and mycelia. Herbaria and personal collections for specimen deposits are as: Field Museum of Natural History (F); University of California, Berkeley (UC); Cryptogamic Herbarium, Kunming Institute of Botany (HKAS); Royal Botanic Gardens, Kew (KM); University of Tennessee Herbarium (Tenn); T.D. Bruns (TDB) and Bradley R. Kropp (Bkr).

Table S2 Comparison of two calibration scenarios of the ECM fossil with other studies. Node number and divergence dates were referred from step one calibration in Figure S3.

Fig. S1 *Suillus* phylogeny of global ITS sequences from both sporocarps and environmental samples. Hosts and geographic information are annotated with taxa names. Taxa with stars are for generating the multigene datasets. Nodes highlighted in black bold bars are supported by both ≥70% bootstraps of maximum likelihood (MLB) and ≥0.98 posterior probability of Bayesian analysis (BPP), annotated above as "MLB / BPP". Grey bared nodes are supported by either MLB or BPP.

Fig. S2 *Suillus* phylogeny of concatenated 28S, and exon regions of *TEF1*, *RPB1* and *RPB2*. Subgenera names are annotated by the terminal taxa. Nodes highlighted in black bold bars are supported by both MLB and BPP, annotated above as "MLB / BPP". Grey bared nodes are supported by either MLB or BPP.

Fig. S3 Step one calibration of the Agaricomycetes phylogeny with two fossils. Both panels were calibrated with the Marasmioid fossil at node 3. Panel A was calibrated under the Suillineae scenario with the ECM fossil at node A. Panel B was under the *Suillus-Pinus* scenario with the ECM fossil at node G. Blue bars at the nodes represent 95% HPD and numbers indicate the median ages. Red nodes are supported by both ≥ 70% MLB and ≥ 0.98 BPP and cyan nodes are supported by either MLB or BPP. Divergence dates were compared in table S2.

Fig. S4 Systematic evolution and diversification of *Suillus* from step two calibration of the *Suillus* phylogeny under the Suillineae scenario. Current host association for each terminal taxon is labeled. Blue bars at the nodes represent 95% HPD and

numbers indicate the median ages. Ancestral host associations are labeled on the nodes: pie charts on the left are from MCMC analysis and on the right from maximum likelihoods. *Larix* associations are labeled in green, *Pseudotsuga* associations are in blue, *Pinus* subgenus *Pinus* associaions are in yellow, *Pinus* subgenus *Strobus* associations are in purple, red demarks an association with *Quercus* after prior establishment with subgenus *Strobus*, and unknown associations are in gray. Red nodes are supported by both ≥ 70% MLB and ≥ 0.98 BPP and cyan nodes are supported by either MLB or BPP.

Fig. S5 Step two calibration of the *Suillus* phylogeny under the *Suillus-Pinus* scenario and ancestral host reconstruction. Current host association for each terminal taxon is labeled. Blue bars at the nodes represent 95% HPD and numbers indicate the median ages. Ancestral host associations are labeled on the nodes: pie charts on the left are from MCMC analysis and on the right from maximum likelihoods. *Larix* associations are labeled in green, *Pseudotsuga* asociations are in blue, *Pinus* subg. *Pinus* associaions are in yellow, *Pinus* subg. *Strobus* associations are in purple, red demarks an association with *Quercus* after prior establishment with subg. *Strobus*, and unknown associations are in gray. Red nodes are supported by both ≥ 70% MLB and ≥ 0.98 BPP and cyan nodes are supported by either MLB or BPP.

Fig. S6 Historical biogeography of Pinaceae estimated by DEC+J model in BioGeoBEARS. Pie diagrams at each node denote geographical units or combination of units occupied by ancestral taxa. Geographic units are represented by different colors, and combined units are shown by hatching colors or by lettering. Width of pie wedges refers to the probability of that geographic unit or combination of units. White wedges indicate the sum of units (or combined units) with individual probabilities <15%. *Suillus* associations were annotated as vectors by the nodes. Current geographic ranges of terminal taxa are indicated by colored boxes and lettering. Parentheses annotate genera, and sections and subgenera are named under the corresponding branches.

Fig. S7 Lineage through time plot of *Suillus*.

Fig. S8 Phylorate plot of *Suillus* with branches colored by speciation rate (lineages/ Ma) as indicated by the scale bar of the Bayesian analysis of macro-evolutionary mixtures (BAMM) analysis

Table S1. Herbaria specimens and molecular sequences used in this study. GenBank sequences are underlined. Host information is included in this table inferred from environmental samples containing root tip and mycelia. Herbaria and personal collections for specimen deposits are as: Field Museum of Natural History (F); University of California, Berkeley (UC); Cryptogamic Herbarium, Kunming Institute of Botany (HKAS); Royal Botanic Gardens, Kew (KM); University of Tennessee Herbarium (Tenn); T.D. Bruns (TDB) and Bradley R. Kropp (Bkr).

Taxonomic name	Herbarium number	Collection number	ITS	28S	*TEF1*	*RPB1*	*RPB2*	Host	Host identifica-tion	Collection site
Suillus acidus		BHS200999	GU188434							USA, Massachusetts
Suillus acidus	TENN066904		KU721166	KU721522	KU721708	KU852235	KU852347			USA, Tennessee
Suillus alpinus	HKAS63128	Shi697	KX342857	KU663237	KU721665	KU852257	KU852328	*Larix potaninii*	field observation	China, Yunnan
Suillus amaranthii		Saylor-4236	U74615					*Pinus lambertiana*	field observation	USA, California
Suillus americanus		WCG2494	KU663184		KU663205	KU852261	KU852351	*Pinus strobus*	field observation	USA, Indiana
Suillus americanus	F1187271		KU663181		KU663203		KU852354	*Pinus strobus*	field observation	USA, Indiana
Suillus asiaticus		JV-4850F	L54090					*Larix laricina*	field observation	USA, Michigan
Suillus asiaticus	HKAS63145	Shi579	KU721410	KU721248	KU721568	KU852262	KU852369	*Larix gmelinii*	field observation	China, Heilongjiang
Suillus aurihymenius	HKAS63130	Shi616	KX342859	KU663242	KU721658	KU852265	KU852326	*Larix gmelinii*	field observation	China, Neimengu
Suillus bellinii		E84	HM545734					*Pinus pinaster*	mycelia	Italy
Suillus bellinii	KM143046		KU721183	KU721352	KU721635	KU852266	KU852287			Italy
Suillus bovinus		68/08	FJ816753					*Pinus pinaster*	root tip	Spain
Suillus bovinus		BP482	FN678890					*Pinus sylvestris*	root tip	Czech Republic
Suillus bovinus	KM164971		KU721203	KU721292	KU721733	KU852269	KU852336			England
Suillus bovinus	HKAS63164	Shi587	KU721196	KU721290	KU721730					China, Heilongjiang
Suillus bresadolae	HKAS63245	Shi605	KU721449	KU663218	KU721687			*Larix gmelinii*	field observation	China, Heilongjiang
Suillus brevipes			EF619768					*Pinus taeda*	mycelia	USA, North Carolina

Taxonomic name	Herbarium number	Collection number	ITS	28S	*TEF1*	*RPB1*	*RPB2*	Host	Host identifica-tion	Collection site
Suillus brevipes			HQ257510					*Pinus banksiana*	root tip	
Suillus brevipes		MGW-1992	L54091					*Pinus* subgenus *Pinus*	field observation	USA, Minnesota
Suillus brevipes	UC1860327			KU721322	KU721761					USA, California
Suillus brevipes	F1187371		KU721224	KU721323	KU721760	KU852270	KU852302			Canada, Alberta
Suillus c.f. americanus		SS5314	JN119753					*Pinus wallichiana*	root tip	Pakistan
Suillus c.f. americanus			HM044608					*Pinus cembra*	root tip	Italy
Suillus c.f. americanus	YNP2355		KU663196		KU663204					USA, California
Suillus c.f. americanus	HKAS91415	RZ07291202	KU663189	KU721520	KU663197	KU852259	KU852349	*Pinus armandii*	field observation	China, sichuan
Suillus c.f. americanus	HKAS57105	Feng376	KU663186		KU663202					China, Tibet
Suillus c.f. americanus	HKAS63155	Shi494	KU663190		KU663198	KU852258	KU852348			China
Suillus c.f. brevipes			JQ711940					*Pinus contorta*	mycelia	Canada, British columbia
Suillus c.f. brevipes			JQ310820					*Pinus muricata*	root tip	USA, California
Suillus c.f. cavipes		IKE41.2	AB284452							Japan
Suillus c.f. cavipes 3	HKAS63149	Shi593	KU721543	KU721413	KU721575			*Larix gmelinii*	field observation	China, Heilongjiang
Suillus c.f. cavipes	HKAS71862	Shi713	KU721546	KU721423	KU721576			*Larix potaninii*	field observation	China, Sichuan
Suillus c.f. cavipes			HM044472					*Larix laricina*	root tip	Italy
Suillus c.f. cavipes 2	HKAS63161	Shi610	KU721544	KU721415	KU721574			*Larix gmelinii*	field observation	China, Heilongjiang
Suillus c.f. collinitus		VC–1042	L54121							Nepal
Suillus c.f. collinitus		BP89	FN679006					*Pinus strobus*	root tip	Czech Republic
Suillus c.f. collinitus		ScG1	JQ685733					*Pinus heldreichii*	mycelia	Montenegro
Suillus c.f. collinitus	HKAS71782	Shi266	KU721182	KU721350						China, Yunnan

Taxonomic name	Herbarium number	Collection number	ITS	28S	*TEF1*	*RPB1*	*RPB2*	Host	Host identification	Collection site
Suillus c.f. collinitus		Aww521	KU721181	KU721351	KU721614		KU852294			China, Yunnan
Suillus c.f. flavidus		PT_31	FJ660477					*Pinus albicaulis*	root tip	USA, Montana
Suillus c.f. granulatus 2		P09049	AB587774					*Pinus thunbergii*	root tip	South Korea
Suillus c.f. granulatus 2	HKAS71922	Shi828	KU721235	KU721340		KU852195	KU852296	*Pinus tabuliformis*	field observation	China, Shandong
Suillus c.f. granulatus 2	HKAS63209	Shi445	KU721233	KU721341	KU721617	KU852194		*Pinus tabuliformis*	field observation	China, Jilin
Suillus c.f. luteus	HKAS63212	Shi690	KU721214	KU721393	KU721602	KU852247	KU852310			China, Yunnan
Suillus c.f. luteus	HKAS72004	Shi1032	KU721217	KU721396	KU721605					China, Yunnan
Suillus c.f. paluster	HKAS63135	Shi599	KU721254	KU721408	KU721582			*Larix gmelinii*	field observation	China, Heilongjiang
Suillus c.f. pinetorum		P09013	AB587773					*Pinus thunbergii*	root tip	South Korea, Kangwon-do
Suillus c.f. pinetorum		AA-1	AB571499					*Pinus densiflora*	root tip	South Korea, Hongcheon
Suillus c.f. pinetorum	HKAS63200	Shi393	KU721198	KU721295	KU721732	KU852268				China, Guizhou
Suillus c.f. pinetorum	HKAS63137	Shi344	KU721195	KU721294	KU721729	KU852267		*Pinus massoniana*	field observation	China, Hunan
Suillus c.f. placidus		PT-51	FJ660478					*Pinus albicaulis*	mycelia	USA, Montana
Suillus c.f. plorans	HKAS63243	Shi664	KU721159	KU721317	KU721740	KU852278		*Pinus armandii*	field observation	China, Shanxi
Suillus c.f. plorans	HKAS71893	Shi763	KU721160	KU721318	KU721741	KU852279		*Pinus armandii*	field observation	China, Hubei
Suillus c.f. plorans 2	HKAS63234	Shi558	KU721163	KU721316	KU721739	KU852277		*Pinus koraiensis*	field observation	China, Heilongjiang
Suillus caerulescens	HKAS93543	RZ120207	KU721188	KU721358	KU721632	KU852272	KU852375	*Pseudotsuga menziesii*	field observation	USA, California
Suillus cavipes Asia			FJ803948					*Larix gmelinii*	root tip	China, inner Mongolia
Suillus cavipes Asia	HKAS63146	Shi598	KU721541	KU721411	KU721571	KU852274		*Larix gmelinii*	field observation	China, Heilongjiang
Suillus cavipes NA		TDB646	KU721548	KU721428	KU721572	KU852276	KU852368	*Larix laricina*	field observation	USA, south Michigan

Taxonomic name	Herbarium number	Collection number	ITS	28S	*TEF1*	*RPB1*	*RPB2*	Host	Host identifica-tion	Collection site
Suillus collinitus		ECM 14	AY953421					*Pinus halepensis*	root tip	Spain
Suillus collinitus		J3.15.2	L54089							France
Suillus collinitus	KM169468			KU721353	KU721616		KU852290			France
Suillus cothurnatus		NSW4662	L54092							USA, Louisiana
Suillus cothurnatus		126B3	JX017266					*Pinus caribaea*	root tip	USA, Puerto Rico
Suillus cothurnatus		JLP2510	DQ377410					*Pinus taeda*	mycelia	USA, North Carolina
Suillus cothurnatus		WT	AM502982					*Pinus sylvestris*	root tip	Austria, South-Eastern Queensland
Suillus decipiens	HKAS93525	BKr1029002	KU721495	KU721521	KU721709		KU852362	*Pinus caribaea*	field observation	Belize
Suillus flavidus		BJP38T_200	GU289423					*Pinus sylvestris*	root tip	Scotland
Suillus flavidus	KM171907		KU721177	KU721378	KU721707					Scotland
Suillus flavipunctipes		RFLP_871	GQ240922					*Pinus massoniana*	root tip	China, Sichuan
Suillus flavipunctipes		RZ08201207	KX342861	KU721336	KU721624	KU852282	KU852298	*Pinus yunnanensis*	field observation	China, Yunnan
Suillus flavipunctipes	HKAS71974		KU721239	KU721335	KU721625					China, Guangdong
Suillus fluryi	KM126760		KU721180	KU721349			KU852293			England
Suillus fuscotomentosus	C0300855F	BW6	KU721158	KU721309	KU721747	KU852283	KU852340			USA, California
Suillus fuscotomentosus		RZ120204	KU721155	KU721307	KU721743	KU852284	KU852337	*Pinus radiata*	field observation	USA, California
Suillus fuscotomentosus		TDB3795	KU721153	KU721310	KU721744		KU852338	*Pinus ponderosa*	field observation	USA, California
Suillus glandulosipes			HQ257494					*Pinus banksiana*	root tip	
Suillus glandulosipes		TDB–3050	KU721225	KU721325	KU721759	KU852286	KU852301	*Pinus banksiana*	field observation	USA, California
Suillus granulatus		D3	HQ439177					*Pinus densiflora*	root tip	China
Suillus granulatus		BP107	FN679003					*Pinus sylvestris*	root tip	Czech Republic

Taxonomic name	Herbarium number	Collection number	ITS	28S	*TEF1*	*RPB1*	*RPB2*	Host	Host identifica-tion	Collection site
Suillus granulatus		SGG1	JQ685727					*Pinus heldreichii*	root tip	Montenegro
Suillus granulatus	KM172141		KU721242	KU721344	KU72163 7		KU8522 95			Italy
Suillus granulatus	HKAS63250	Shi649	KU721241							china, Heilongjiang
Suillus greivillei Asia	HKAS63206	Shi604	KU721480	KU663265	KU72165 4	KU8522 29	KU8523 20	*Larix gmelinii*	field observation	China, Heilongjiang
Suillus greivillei NA		TDB570	KU721489	KU663262	KU72163 4	KU8522 34	KU8523 78	*Larix laricina*	field observation	USA, south Michigan
Suillus grevillei Euro			HM044473					*Larix decidua*	root tip	Italy
Suillus grevillei Euro	KM172554		KU721486	KU663249	KU72165 3	KU8522 32	KU8523 24	*Larix decidua*	field observation	England
Suillus grisellus		TDB574	KU721464	KU663234	KU72168 0	KU8522 16	KU8523 29	*Larix laricina*	field observation	USA, south Michigan
Suillus grisellus	HKAS63241	Shi627	KU721448	KU663217	KU72167 6	KU8522 09		*Larix gmelinii*	field observation	China, Neimengu
Suillus hirtellus		1S3.18.S05	EF619770					*Pinus taeda*	mycelia	USA, North Carolina
Suillus hirtellus	TENN064576		KU721152	KX171000	KX17100 4		KX1710 07			USA, Tennessee
Suillus kwangtungensis	HKAS71979	Shi978	KU721518	KU721539	KU72171 0	KU8522 39		*Pinus kwangtungensis*	field observation	China, Guangdong
Suillus kwangtungensis	HKAS90666	Rui364	KU721540	KU721519	KU72171 1	KU8522 40		*Pinus kwangtungensis*	field observation	China, Guangdong
Suillus lakei		EM8	JX003290					*Pseudotsuga menziesii*	root tip	Canada
Suillus lakei		RZ120208	KU721189	KU721361	KU72158 9	KU8522 42	KU8523 74	*Pseudotsuga menziesii*	field observation	USA, California
Suillus luteus			GQ985411					*Pinus tabulaeformis*	root tip	China, Beijing
Suillus luteus			AB211142					*Pinus densiflora*	root tip	
Suillus luteus			FN691763					*Pinus sylvestris*	root tip	Czech Republic
Suillus luteus			AB506096					*Pinus thunbergii*	root tip	South Korea
Suillus luteus			AB839396					*Pinus massoniana*	root tip	Japan
Suillus luteus	KM171470		KU721218	KU721399			KU8523 11			England

Taxonomic name	Herbarium number	Collection number	ITS	28S	*TEF1*	*RPB1*	*RPB2*	Host	Host identification	Collection site
Suillus luteus		PRL11285	KU721219	KU721398	KU721606	KU852248	KU852308			USA
Suillus c.f. marginielevatus	HKAS63238	Shi702	KX342860	KU721320	KU721725	KU852219	KU852367	*Pinus armandii*	field observation	China, Yunnan
Suillus c.f. marginielevatus	HKAS63237	Shi701	KU721169	KU721319	KU721727	KU852218		*Pinus armandii*	field observation	China, Yunnan
Suillus c.f. marginielevatus	HKAS63254	Shi703	KU721168	KU721321	KU721726	KU852220		*Pinus armandii*	field observation	China, Yunnan
Suillus mediterraneensis		ECM 67	AJ410860					*Pinus halepensis*	root tip	France
Suillus mediterraneensis		JAM0750	HM347657					*Pinus halepensis*	field observation	Spain
Suillus megaporinus	UC1860326		KU721178	KU721379	KU721701					USA, California
Suillus mimicus	HKAS63176	Shi696	KU721438	KU663211	KU721693	KU852243	KU852332	*Larix potaninii*	field observation	China, Yunnan
Suillus occidentalis		HDT28175	GQ249394					*Pinus ponderosa*	root tip	USA, Arizona
Suillus occidentalis			JN704816					*Pinus montezumae*	root tip	Mexico
Suillus ochraceoroseus	F1186906		KU721258	KU721404	KU721584	KU852250		*Larix occidentalis*	field observation	USA, Idaho
Suillus ochraceoroseus	HKAS63134	Shi600	KU721255	KU721407	KU721586	KU852251	KU852371	*Larix gmelinii*	field observation	China, Heilongjiang
Suillus phylopictus		Gifu17.2	AB284466					*Pinus pumila*	mycelia	Japan, Gifu
Suillus phylopictus	HKAS63184	Shi446	KU721535	KU721514	KU721714			*Pinus koraiensis*	field observation	China, Jilin
Suillus phylopictus	HKAS91411	RZ07281202	KU721529	KU721508	KU721715	KU852254	KU852361	*Pinus armandii*	field observation	China, Yunnan
Suillus phylopictus	HKAS90664	Rui362	KU721538	KU721517	KU721712	KU852253		*Pinus kwangtungensis*	field observation	China, Guangdong
Suillus pinetorum		RZ08151203	KU721208	KU721300	KU721736	KU852188	KU852334	*Pinus yunnanensis*	field observation	China, Yunnan
Suillus pinetorum		RZ07231201	KU721205	KU721297	KU721734	KU852186	KU852333	*Pinus yunnanensis*	field observation	China, Yunnan
Suillus placidus		kh14	KF209350					*Pinus cembra*	Mesh bag	Austria
Suillus placidus	TENN062310		KU721274	KU721389	KU721558	KU852189	KU852364			USA, Massachusetts
Suillus placidus	F1112674		KU721260	KU721381	KU721559		KU852363			USA, Michigan

Taxonomic name	Herbarium number	Collection number	ITS	28S	*TEF1*	*RPB1*	*RPB2*	Host	Host identifica-tion	Collection site
Suillus plorans	KM104747		KU721164	KU721312	KU721737			*Pinus cembra*	field observation	Italy
Suillus plorans	HKAS63225	Shi588	KU721165	KU721313	KU721738					China, Heilongjiang
Suillus ponderosus		RZ120201	KU721187	KU721364	KU721590	KU852190	KU852376	*Pseudotsuga menziesii*	field observation	USA, California
Suillus pseudobrevipes		TKDN290	AY587754					*Pinus jeffreyi*	root tip	USA, California
Suillus pseudobrevipes			AY880938					*Pinus contorta*	root tip	USA, California
Suillus pseudobrevipes			FJ197214					*Pinus muricata*	root tip	USA, California
Suillus pseudobrevipes		YNP2390	KU721229	KU721329	KU721612	KU852191	KU852305	*Pinus ponderosa*	field observation	USA, California
Suillus punctipes		TDB–265	L54098							USA, Minnesota
Suillus pungens			JN704830					*Pinus montezumae*	root tip	Mexico
Suillus pungens			JQ917859					*Pinus radiata*	root tip	USA, California
Suillus pungens			JQ310819					*Pinus muricata*	root tip	USA, California
Suillus pungens			DQ351504					*Pinus jeffreyi*	root tip	USA, California
Suillus pungens		RZ120202	KX342862	KU721326	KU721630	KU852196	KU852299	*Pinus radiata*	field observation	USA, California
Suillus quiescens		9_ENYO_j_19	DQ351501					*Pinus jeffreyi*	root tip	USA, California
Suillus quiescens		TG90	GU301274					*Pinus contorta*	root tip	USA, Oregon
Suillus quiescens		DISP7	JN858077					*Pinus muricata*	root tip	USA, California
Suillus quiescens		R3190	EF458012					*Pinus ponderosa*	root tip	USA, Oregon
Suillus quiescens	C0300858F	RZ120206	KU721184	KU721354	KU721756	KU852198	KU852288			USA, California
Suillus salmonicolor	HKAS93524	BKr1029001	KX170996	KX170999	KX171003		KX171006	*Pinus caribaea*	field observation	Belize
Suillus serotinus	TENN061628		KU721465	KU663235	KU721679	KU852217	KU852330	*Larix laricina*	field observation	Canada, newfoundland
Suillus sp. 2	HKAS71980	Shi979	KU721491					*Pinus kwangtungensis*	field observation	China, Guangdong

Taxonomic name	Herbarium number	Collection number	ITS	28S	*TEF1*	*RPB1*	*RPB2*	Host	Host identification	Collection site
Suillus sp. 3			AB284470					*Pinus koraiensis*	root tip	Japan
Suillus sp. 3		Ontake13B	AB284469					*Pinus koraiensis*	root tip	Japan, Nagano
Suillus sp. 3		TakamineW	AB284473					*Pinus parviflora*	root tip	Japan, Gunma
Suillus sp. 3		Kusatsu6W1	AB284455					*Pinus pumila*	root tip	Japan, Gunma
Suillus sp. 3			HM044533					*Larix laricina*	root tip	Italy
Suillus sp. 3			HM044567					*Larix laricina*	root tip	Italy
Suillus sp. 3	HKAS29529		KU721498	KU721373	KU721704					China
Suillus sp. 3	HKAS71797	Shi284	KU721500	KU721375	KU721703	KU852204	KU852346			China, Yunnan
Suillus sp. 4	HKAS63240	Shi615	KU721497							China, Heilongjiang
Suillus sp. 4	HKAS63222	Shi601	KU721496							China, Heilongjiang
Suillus spectabilis		TDB641	KU721556	KU721429	KU721596	KU852221	KU852313	*Larix laricina*	field observation	USA, Michigan
Suillus spectabilis	HKAS63159	Shi591	KU721551	KU721414		KU852223	KU852316	*Larix gmelinii*	field observation	China, Heilongjiang
Suillus spraguei		Aftol ID 717	AY854069	NG_027637	AY883429	AY858965	AY786066	*Pinus strobus*	field observation	USA, Massachusetts
Suillus subalutaceus		ACAD15288	L54075							Canada, Scotia Nova
Suillus subaureus Asia	HKAS56316	Li1476	KU721172	KU721367	KU721694					China, Yunnan
Suillus subaureus Asia	HKAS71798	Shi285	KU721174	KU721369	KU721696					China, Yunnan
Suillus subaureus NA	F1189253		KU721171	KU721365	KU721699	KU852203	KU852356			USA
Suillus subaureus NA	F1187760		KU721170	KU721366	KU721700		KU852355	Broadleaves	field observation	USA, Indiana
Suillus subolivaceus		TDB1492	KU721167					*Pinus monticola*	field observation	USA, California
Suillus tomentosus		FFP848	JQ711881					*Pinus contorta*	root tip	Canada, British columbia
Suillus tomentosus		Li3–2	EF458017					*Pinus resinosa*	root tip	USA

Taxonomic name	Herbarium number	Collection number	ITS	28S	*TEF1*	*RPB1*	*RPB2*	Host	Host identifica-tion	Collection site
Suillus tomentosus	F1186917		KU721157	KU721301	KU721748	KU852205	KU852345			USA, Idaho
Suillus tomentosus	KM169463			KU721306	KU721750		KU852341			England
Suillus tridentinus		HB347	KU721289	KU663244	KU721661	KU852208	KU852325	*Larix decidua*	field observation	West Germany
Suillus umbonatus		FFP496	JQ711850					*Pinus contorta*	root tip	Canada, British columbia
Suillus umbonatus		TDB-978	L54115					*Pinus* subgenus *Pinus*	field observation	USA, California
Suillus umbonatus		S147A	AY880939					*Pinus contorta*	root tip	USA, Oregon
Suillus umbonatus	F1187506		KU721494	KU721377	KU721705		KU852353			Canada, Alberta
Suillus umbrinus		Trappe-7516	U74619					*Pinus monticola*	field observation	USA, California
Suillus variegatus		HB–325	L54081							Germany
Suillus variegatus		P17 F	AM086444					*Pinus nigra*	root tip	Scotland
Suillus variegatus		p10	AJ971401					*Pinus sylvestris*	root tip	Scotland
Suillus variegatus		BP140	FN679001					*Pinus sylvestris*	root tip	Czech Republic
Suillus viscidus			GU181858					*Larix decidua*	root tip	Italy
Suillus viscidus	KM166812		KU721460	KU663230	KU721685	KU852213	KU852331	*Larix decidua*	field observation	England
Suillus viscidus	HKAS71870	Shi726	KU721452	KU663221	KU721686	KU852211		*Larix gmelinii*	field observation	China, Gansu
Suillus weaverae		TDB725	L54108					*Pinus* subgenus *strobus*	field observation	USA, Michigan
Suillus weaverae		TDB878	L54113					*Pinus* subgenus *strobus*	field observation	USA, Michigan
Suillus weaverae		RZH104	KX170995					*Pinus* subgenus *strobus*	field observation	USA, Wisconsin

Table S2 Comparison of two calibration scenarios of the ECM fossil with other studies. Node number and divergence dates were referred from step one calibration in Figure 2.

		Calibration scenario: Suillineae			Calibration scenario: subg. *Suillus*			Floudas et al. 2012		Wilson et al. 2012		Feng et al. 2012		Wilson et al. 2017	
Node	tMRCA	Mean	Median	95% HPD	Mean	Median	95% HPD	Mean	95% HPD	Median	95% HPD	Mean	95% HPD	Mean	95% HPD
1	Root - Agaricomycetes	161.5	159.6	112.8-262.9	303.3	299.2	237.7-360.7	139.6	109.9-176.7					145.8	117.7-179.2
2	Agaricales	137.8	136.2	105.0-226.9	173.8	171.3	134.5-217.7	115.5	94.6-144.8					115.1	93.5-144.4
3	Mycenaceae	95.5	94.1	90.1-144.5	102.5	98.5	90.2-126.9			96.8	90-117.86	134.8	76.8-196.1	101.8	90.2-125.2
4	Boletales	152.1	150.5	119.8-188.7	280.1	279.5	220.2-337.5	97.6	70-129.4	128.1	91.6 - 178.7	189	128.8-254.6	101.7	71.6-136.7
5	Sclerodermatineae	81.6	80.8	33.6-69.9	140.6	139.3	97.3-184.5			82.5	54.7-115.4				
6	Boletineae	57.5	56.8	39.8-76.9	97.1	95.7	63.9-133.2								
A	Suillineae	59.9	58.5	50.8-72.3	104.7	103.1	81.2-132.0			56.1	50-74.6				
C	*Suillus*	40.8	40.2	31.9-50.8	72.1	71.1	60.6-85.7								
D	subg. *Boletinus*	28.7	28.4	19.3-38.3	49.8	49.5	32.9-66.2								
E	G + F	36.4	35.9	28.2-45.5	64.9	64	55.8-76.0								
F	subg. *Spectabilis*	28.4	28.2	19.15-38.17	50.5	50.6	34.7-65.9								
G	H + I	30.4	30	23.7-38.5	55.2	54.3	50.4-62.2								
I	subg. *Suillus*	24.4	24.1	18.6-30.9	43.1	42.9	34.6-51.7								
M	Sect. *Diversipes**	24.9	24.6	21.0-29.8	43.1	42.8	36.7-50.6								
L	Sect. *Suillus**	25.0	24.8	21.0-30.1	43.1	42.8	36.2-50.7								
H	J + K	25.8	25.5	18.9-33.3	46.4	46.5	36.2-56.3								
J	subg. *Larigni*	15.5	15.2	10.0-21.2	27.2	26.9	17.7-37.1								
K	subg. *Douglasii*	11.6	11.2	5.8-18	20	19.4	9.4-32.2								

*Only origin dates of the two sections were inferred from step two calibration.

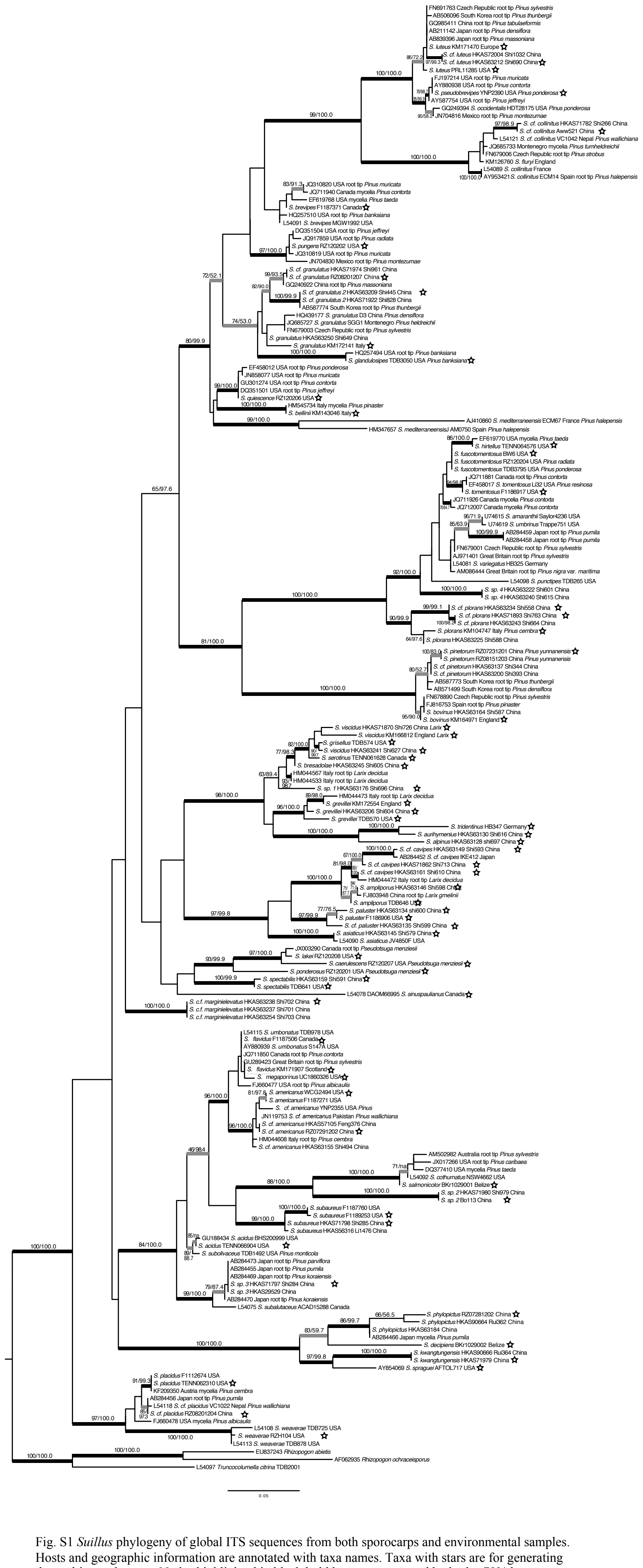

Fig. S1 *Suillus* phylogeny of global ITS sequences from both sporocarps and environmental samples. Hosts and geographic information are annotated with taxa names. Taxa with stars are for generating the multigene datasets. Nodes highlighted in black bold bars are supported by both ≥70% bootstraps of maximum likelihood (MLB) and ≥0.98 posterior probability of Bayesian analysis (BPP), annotated above as “MLB / BPP”. Grey bared nodes are supported by either MLB or BPP.

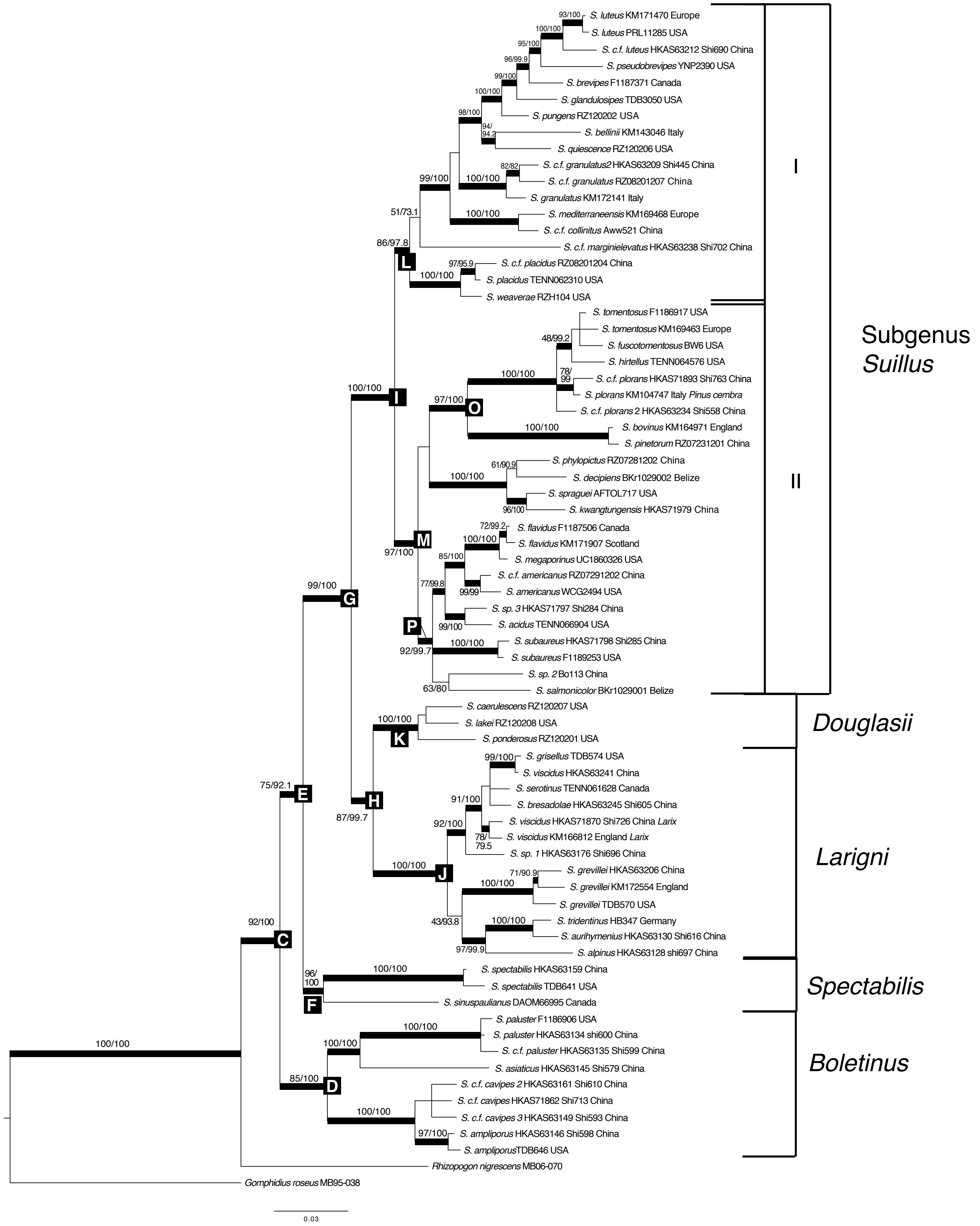

Fig. S2 *Suillus* phylogeny of concatenated 28S, and exon regions of *TEF1*, *RPB1* and *RPB2*. Subgenera names are annotated by the terminal taxa. Nodes highlighted in black bold bars are supported by both MLB and BPP, annotated above as "MLB / BPP". Grey bared nodes are supported by either MLB or BPP.

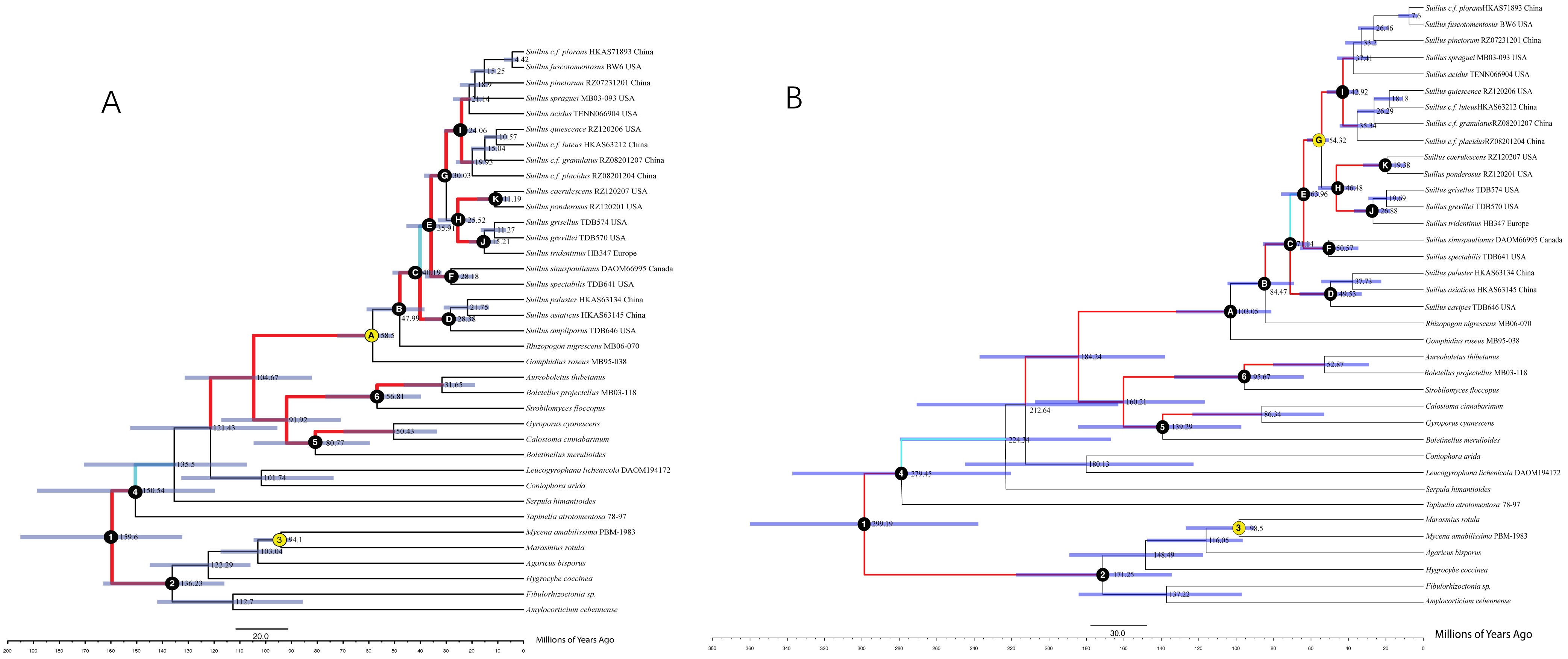

Fig. S3 Step one calibration of the Agaricomycetes phylogeny with two fossils. Both panels were calibrated with the Marasmioid fossil at node 3. Panel A was calibrated under the Suillineae scenario with the ECM fossil at node A. Panel B was under the *Suillus-Pinus* scenario with the ECM fossil at node G. Blue bars at the nodes represent 95% HPD and numbers indicate the median ages. Red nodes are supported by both ≥ 70% MLB and ≥ 0.98 BPP and cyan nodes are supported by either MLB or BPP. Divergence dates were compared in table 1.

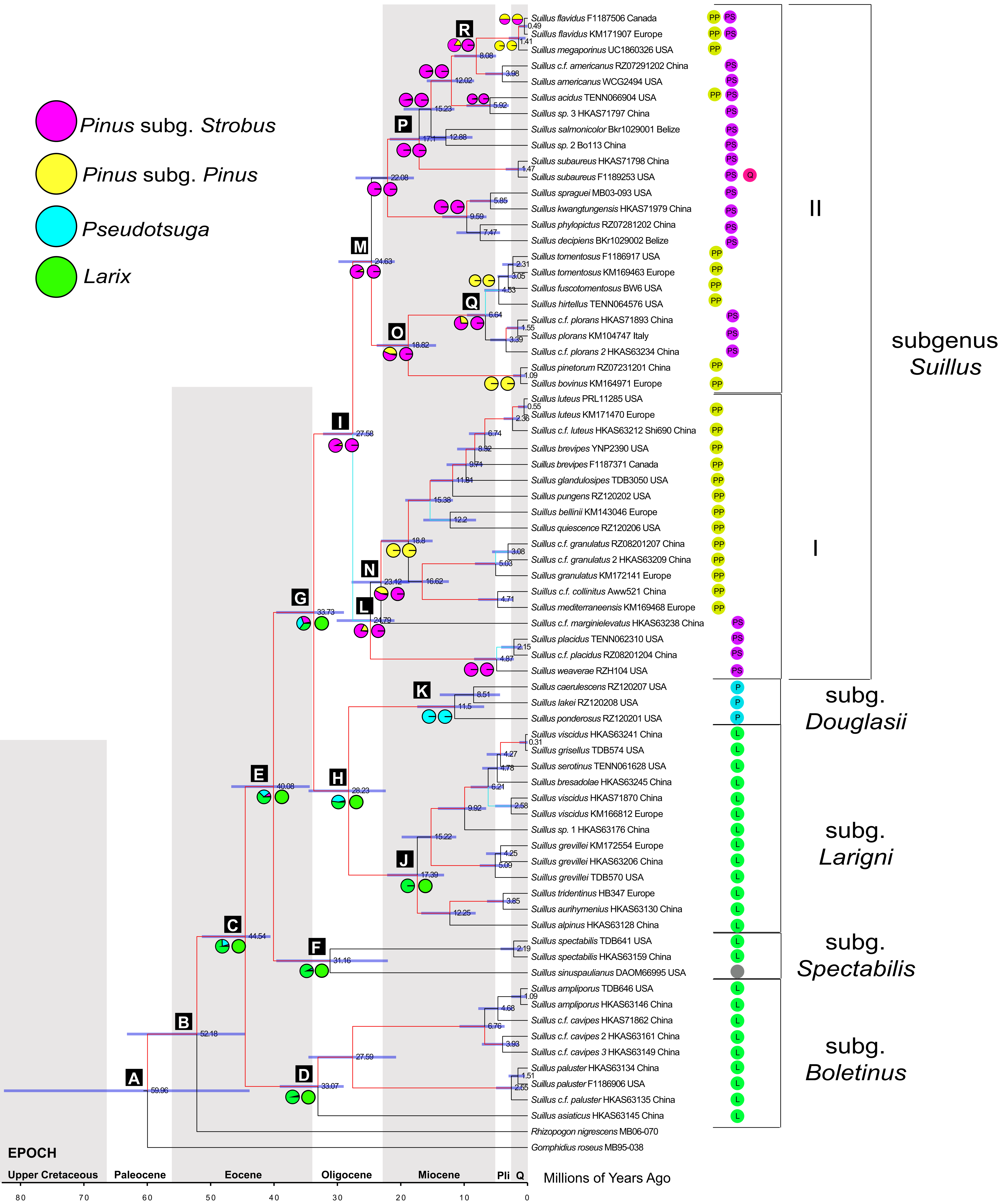

Fig. S4 Systematic evolution and diversification of *Suillus* from step two calibration of the *Suillus* phylogeny under the Suillineae scenario. Current host association for each terminal taxon is labeled. Blue bars at the nodes represent 95% HPD and numbers indicate the median ages. Ancestral host associations are labeled on the nodes: pie charts on the left are from MCMC analysis and on the right from maximum likelihoods. *Larix* associations are labeled in green, *Pseudotsuga* associations are in blue, *Pinus* subgenus *Pinus* associaions are in yellow, Pinus subgenus *Strobus* associations are in purple, red demarks an association with *Quercus* after prior establishment with subgenus *Strobus*, and unknown associations are in gray. Red nodes are supported by both ≥ 70% MLB and ≥ 0.98 BPP and cyan nodes are supported by either MLB or BPP.

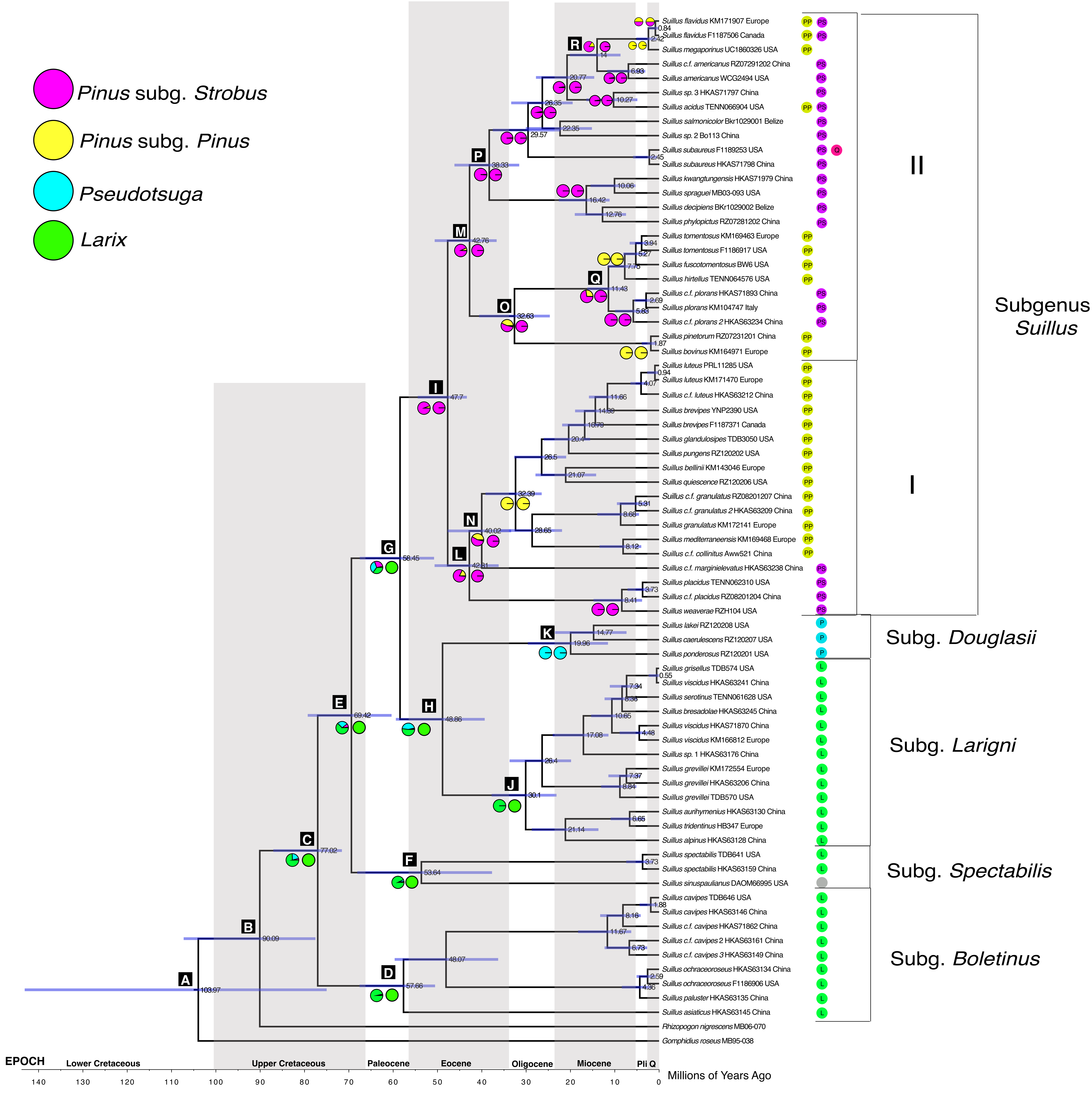

Fig. S5 Step two calibration of the *Suillus* phylogeny under the *Suillus-Pinus* scenario and ancestral host reconstruction. Current host association for each terminal taxon is labeled. Blue bars at the nodes represent 95% HPD and numbers indicate the median ages. Ancestral host associations are labeled on the nodes: pie charts on the left are from MCMC analysis and on the right from maximum likelihoods. *Larix* associations are labeled in green, *Pseudotsuga* associations are in blue, *Pinus* subg. *Pinus* associaions are in yellow, *Pinus* subg. *Strobus* associations are in purple, red demarks an association with *Quercus* after prior establishment with subg. *Strobus*, and unknown associations are in gray. Red nodes are supported by both ≥ 70% MLB and ≥ 0.98 BPP and cyan nodes are supported by either MLB or BPP.

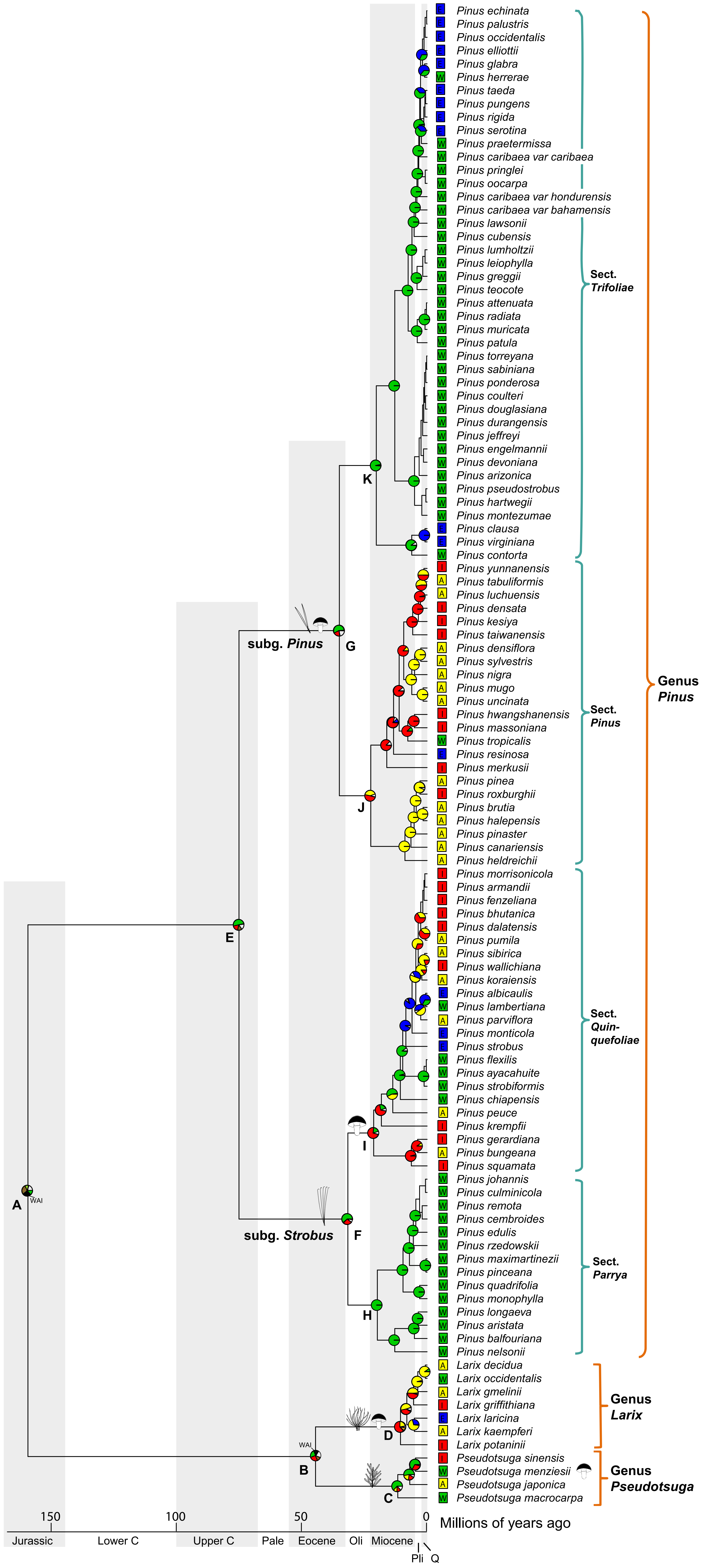

Fig. S6 Historical biogeography of Pinaceae estimated by DEC+J model in BioGeoBEARS. Pie diagrams at each node denote geographical units or combination of units occupied by ancestral taxa. Geographic units are represented by different colors, and combined units are shown by hatching colors or by lettering. Width of pie wedges refers to the probability of that geographic unit or combination of units. White wedges indicate the sum of units (or combined units) with individual probabilities <15%. *Suillus* associations were annotated as vectors by the nodes. Current geographic ranges of terminal taxa are indicated by colored boxes and lettering. Parentheses annotate genera, and sections and subgenera are named under the corresponding branches.

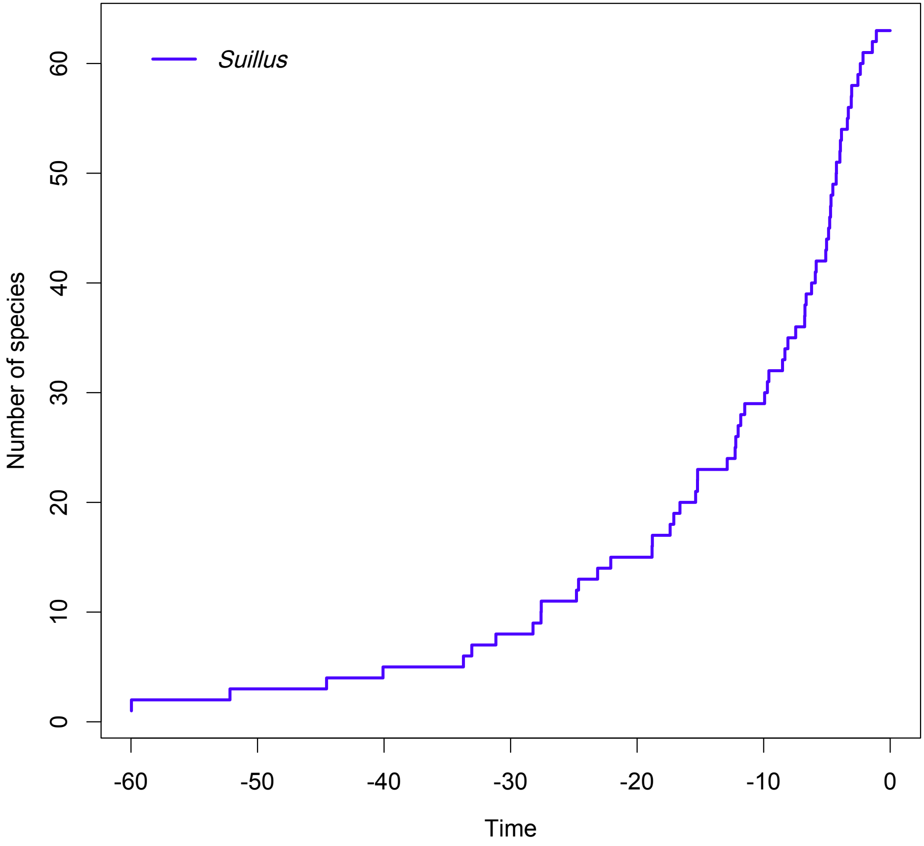

Fig. S7 Lineage through time plot of *Suillus*

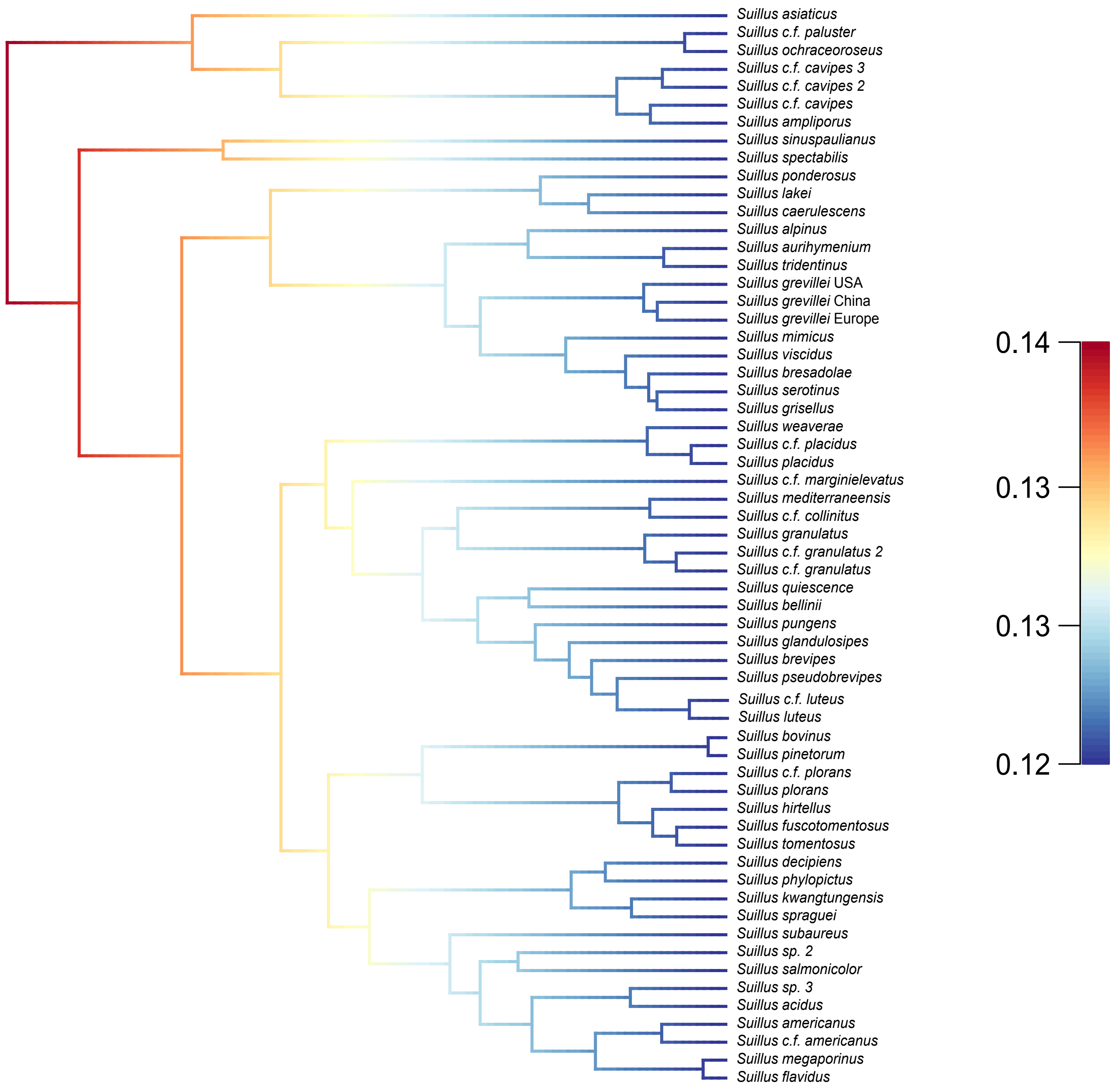

Fig. S8 Phylorate plot of *Suillus* with branches colored by speciation rate (lineages/ Ma) as indicated by the scale bar of the Bayesian analysis of macro-evolutionary mixtures (BAMM) analysis.
